# Development of In-Hospital Outcomes in Patients undergoing Transcatheter Aortic Valve Implantation (TAVI) at an Interdisciplinary Heart Center: A Single-Center Experience of 489 Consecutive Cases

**DOI:** 10.26502/fccm.92920309

**Published:** 2023-03-13

**Authors:** Mukaram Rana, Margit Niethammer, Christian Sellin, Hilmar Dörge, Holger Eggebrecht, Volker Schächinger

**Affiliations:** 1 Herz-Thorax-Zentrum Fulda, Medizinische Klinik I (Kardiologie, Angiologie, Intensivmedizin), Klinikum Fulda, Universitätsmedizin Marburg - Campus Fulda, Fulda; 2 Herz-Thorax-Zentrum Fulda, Klinik für Herz- und Thoraxchirurgie, Klinikum Fulda, Universitätsmedizin Marburg - Campus Fulda, Fulda; 3 Cardiologisches Centrum Bethanien (CCB), Frankfurt a. M.

**Keywords:** Aortic stenosis (AS), In-hospital outcomes, Transcatheter Aortic Valve Implantation (TAVI)

## Abstract

**Background:**

Transcatheter Aortic Valve Implantation (TAVI) has emerged over time, reflected in appropriate adjustments in the European Society of Cardiology (ESC) guidelines in 2007, 2012 and 2017.

**Objective:**

The aim of this study was to analyze in-hospital outcomes after TAVI in the development within a single heart center over a period of 10 years depending on adjustments in the guidelines, infrastructural and procedural determinants.

**Methods:**

489 consecutive patients who underwent TAVI from 2010 and 2019 at our center were analyzed retrospectively. Patients were divided into 3 groups of different treatment circumstances depending on guidelines adjustments and local infrastructural progress (group 1: 2010–2015 (n = 132), group 2: 2016–2017 (n = 155), group 3: 2018–2019 (n = 202). The primary endpoint was defined as all-cause in-hospital mortality. Secondary endpoints were selected according to the Valve Academic Research Consortium (VARC)-2 definitions. Multivariate logistic regression analysis was performed to determine predictors of in-hospital mortality. Statistical significance was assumed for p < 0.05.

**Results:**

489 patients (346 (70.8 %) transfemoral and 143 (29.2 %) transapical) underwent TAVI. Comparing periods (group 1 vs. 2 vs. 3) age (82.1 ± 6.2 vs. 82.5 ± 4.8 vs. 81.1 ± 5.1 years, p = 0.012) and EuroSCORE II (8.4 ± 6.0 vs. 5.8 ± 4.9 vs. 5.5 ± 5.0 %, p < 0.001) declined over time. Rates of in-hospital mortality decreased significantly (9.1 % vs. 5.8 % vs. 2.5 %, p = 0.029), especially with observed-to-expected mortality ratios indicating a disproportionate decline of in-hospital mortality (1.08 vs. 1.00 vs. 0.45). Furthermore, post-procedural complications, such as acute kidney injury stage 3 (10.6 % vs. 3.2 % vs. 4.5 %, p = 0.016) and bleeding complications (14.4 % vs. 11.6 % vs 7.9 %, p = 0.165) decreased from group 1 to 3. However, rates of permanent pacemaker implantations (7.6 % vs. 11.0 % vs. 22.8 %, p < 0.001) increased, associated with a switch towards self-expanding valves (0.0 % vs. 61.3 % vs. 76.7 %, p < 0.001). Length of hospitalization as well as stay at intensive care and intermediate care unit could be reduced significantly during the observation period. In multivariate analysis age (OR: 1.103; 95 % CI: 1.013 – 1.202; p = 0.025), creatinine level before TAVI (OR: 1.497; 95 % CI: 1.013 – 2.212; p = 0.043), atrial fibrillation (OR: 2.956; 95 % CI: 1.127 – 7.749; p = 0.028) and procedure duration (OR: 1.017; 95 % CI: 1.009 – 1.025; p < 0.001) could be identified as independent predictors of in-hospital mortality.

**Conclusion:**

This study identified age, creatinine level before TAVI, the presence of atrial fibrillation and procedure duration as independent predictors for in-hospital mortality. Although these predictors decreased during the observation period, the decline in hospital-mortality was disproportionate, which was indicated by an observed-to-expected mortality ratio of 0.45 for the last observation period. However, it can be assumed that apart from patient-related factors, there were further institutional, technical and procedural developments, which ran in parallel and affected in-hospital mortality rates after TAVI.

## Graphical Abstract

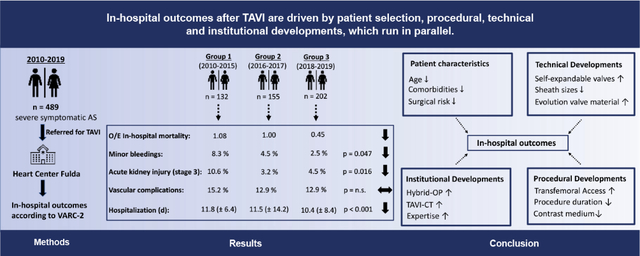

## 1. Introduction

Aortic stenosis (AS) is the third most common cardiovascular disease after coronary artery disease and arterial hypertension and the leading primary valvular heart disease in Europe and North America [1]. As the onset of symptoms is associated with a shortage of life expectancy and a poor outcome, both appropriate diagnosis and treatment are of major importance [2,3]. Since the first Transcatheter Aortic Valve Implantation (TAVI) has been performed by Alain Cribier in 2002, this interventional approach has experienced rapid dissemination and thus has been accepted as viable alternative in the treatment of patients at high and intermediate surgical risk [4,5]. Although remarkable improvements in outcomes after TAVI could be observed over the last years, there is still a paucity of real-world data analyzing the developments, which have influenced clinical results after TAVI. In the Heart-Thorax Center Fulda (Herz-Thorax Zentrum Fulda) the two disciplines performing TAVI, the cardiology department and the cardiac surgery department are closely connected, defining patient care from a single source. In addition, there is a staff continuity over years, developing the TAVI program together. The main objective of this study was to analyze under these conditions the impact of changing guideline recommendations, technical, institutional and procedural developments on in-hospital outcomes after TAVI.

## 2. Material and Methods

### 2.1 Study Design and Population

In this single-center study we retrospectively analyzed data of patients undergoing TAVI at our heart center. From 01.01.2010 until 31.12.2019 a total of 489 consecutive patients with severe symptomatic aortic stenosis were enrolled in this study. Three groups (group I: 2010 – 2015 (n = 132), group II: 2016 – 2017 (n= 155) and group III: 2018 – 2019 (n= 202) were created according to changing ESC guidelines (2007, 2012 and 2017) and institutional developments to compare outcomes between different time periods [5–7]. Patients with symptomatic aortic stenosis requiring a TAVI as decided by the local heart team were eligible for inclusion in this study. Patients with absolute contraindications as defined in the current European Society of Cardiology guidelines were excluded. This retrospective study was approved by the ethics committee at the University of Marburg (ek_mr_010720_rana).

### 2.2 Demographics and Clinical Features

Data were retrieved from our local TAVI registry, discharge reports and internal electronical database. Demographic and clinical data included age, gender, body mass index (BMI), body surface area (BSA), cardiovascular risk factors (hypertension, diabetes mellitus, renal insufficiency, hyperlipidemia, obesity, and smoking) and a history of cardiac decompensation. Severity of symptoms was assessed using the New York Heart Association (NYHA) and the Canadian Cardiovascular Society (CCS) classification. Data of relevant cardiac comorbidities (history of ST-elevation myocardial infarction (STEMI) or Non-ST-elevation myocardial infarction (NSTEMI), coronary heart disease with 1-, 2-, 3- vessel disease, left main artery disease) and non-cardiac comorbidities (peripheral and cerebrovascular disease, chronic obstructive pulmonary disease (COPD), stroke, transient ischemic attack (TIA), history of cancer and anemia) were collected from medical reports and our database, if available. Furthermore, patients were screened for prior cardiac interventions, such as bypass and valve operations, balloon valvuloplasty, percutaneous coronary intervention (PCI) and others (e.g. permanent pacemaker (PPM) implantation). Levels of cardiovascular biomarkers (Troponin T and N-terminal pro-B-type natriuretic peptide, creatinine, hemoglobin, and platelets were measured in blood samples, which were obtained from patients before the procedure.

### 2.3 Preoperative Echocardiography, CT scan and ECG

Preoperative echocardiography was conducted to obtain the left ventricular ejection fraction (LVEF), the mean pressured gradient (MPG), the aortic valve orifice area (AVA) and the aortic annulus diameter. CT scans, which became a mandatory part of the preprocedural planning in 2016, were used to assess the access route (diameters of iliofemoral arteries, presence of kinking, aortic aneurysm, and porcelain aorta), to calculate perimeter- and area-derived diameters of the aortic annulus and the distance of the left and right coronary artery to the aortic annulus. Electrocardiograms (ECGs) were recorded and analyzed on admission and postprocedure. ECGs were screened for rhythm (sinus rhythm or atrial fibrillation (AF)), atrioventricular blocks (degree 1 to 3), left bundle branch block (LBBB), right bundle branch block (RBBB), left anterior fascicular block (LAFB).

### 2.4 Procedural Characteristics

General procedural characteristics of interest comprised procedure duration, contrast medium consumption, fluoroscopy time and radiation dose. Specific procedure-related characteristics were access route (transfemoral or transapical), valve types (balloon-expandable or self-expandable), valve and sheath sizes, predilatation, rapid pacing, postdilatation, conversion to transapical access or surgery, use of cardiopulmonary bypass (CBP) and valve-in-valve procedures.

### 2.5 Postprocedural Outcomes

The primary endpoint was defined as all-cause in-hospital mortality. Following secondary endpoints were selected using the Valve Academic Research Consortium (VARC)-2 definitions [8]: Myocardial infarction, stroke, TIA, bleeding (life-threatening bleeding, major and minor bleeding), acute kidney injury (AKIN stage 1–3 and the need of dialysis), access site and access-related complications (major vascular complication, minor vascular complication and percutaneous closure device failure), conduction disturbances and arrhythmias (new atrioventricular (AV) blocks (degree 1–3), RBBB, LBBB, LAFB, new permanent pacemaker (PPM) implantation, new onset of atrial fibrillation (AF), any new arrhythmia resulting in hemodynamic instability or requiring therapy). Furthermore, valve malposition, ventricle injury, pericardial tamponade, endocarditis, sepsis, paravalvular insufficiency, length of hospitalization and stay at intermediate care unit (IMC) and intensive care unit (ICU) were assessed.

### 2.6 Statistical Analysis

Categorial data are presented as absolute numbers and percentages. The Shapiro-Wilk was used to test the normal distribution for all continuous variables. Continuous variables are expressed as mean (± standard deviation [SD]) and median (interquartile range [IQR]: 25th to 75th percentiles). Categorial variables were compared between the three groups performing pairwise chi-square test. Inter-group comparisons for continuous variables were performed using Kruskal Wallis test or ANOVA as appropriate. Multivariate logistic regression analysis was performed using the enter method to determine independent predictors of in-hospital mortality. Statistical significance was assumed at a p-value less than 0.05. All statistical analyses were performed using IBM SPSS Statistics for Windows, version 26.0 (IBM Corp., Armonk, N.Y., USA).

## 3. Results

### 3.1 Patient Characteristics at Baseline

Demographics, clinical features, and comorbidities at baseline per time interval are provided in Table 1 and 2. From January 2010 to December 2019, a total of 489 consecutive patients with severe AS were enrolled in this study. Patients in group 3 (years 2018–2019) were significantly younger, included more men and presented a significant lower predicted surgical risk as assessed by the EuroSCORE 2. Comorbidities, such as peripheral vascular disease (PVD), cerebrovascular disease (CVD), anemia and a history of cancer were significantly more prevalent in group 1 (years 2010–2015). Patients in group 3 displayed significant lower rates of previous myocardial infarction, prior percutaneous coronary interventions and bypass operations.

### 3.2 Preprocedural Imaging and ECG

The results of preprocedural imaging (echocardiography and CT scan) and ECG are shown in Supplementary Tables 2,3 and 4. Patients in group 2 and 3 had a better left ventricular ejection fraction (LVEF) than patients in group 1. There were significant higher rates of patients with aortic aneurysm and porcelain aorta in group 1.

### 3.3 Preprocedural Blood Examinations

Blood examinations prior to TAVI revealed a significant better renal function (1.6 ± 1.1 vs. 1.2 ± 0.4 vs. 1.3 ± 0.9 mg/dl, p = 0.005) and higher hemoglobin values (12.1 ± 1.9 vs. 12.5 ± 1.7 vs. 12.6 ± 1.8 g/dl, p = 0.009) in group 3. NT-pro-BNP levels were significantly lower in group 3. Results of preprocedural blood examinations can be seen in Supplementary Table 1.

### 3.4 Procedural Characteristics

Procedural outcomes are summarized in Supplementary Table 5. The annual number of TAVI procedures has increased from n = 13 (2010) to n = 112 (2019) (Supplementary Figure 1). All 489 TAVI procedures were performed under general anesthesia (100 %). Over time, a total of 345 (70.6 %) transfemoral TAVI procedures were performed with an increasing frequency from the first to the third period. The use of self-expandable transcatheter heart valves increased significantly during the observation period requiring smaller sheath sizes. Procedure duration and laboratory time decreased over time and were significantly lower in group 3. Likewise, contrast volume consumption could be reduced significantly (136.0 ± 66.7 vs. 167.6 ± 84.6 vs. 119.6 ± 56.7 ml, p < 0.001). Rates of conversion to open surgery during TAVI were rare and showed a downward trend from 1.5 % (group 1) to 0.5 % (group 3).

### 3.5 Postprocedural Outcomes

**Figure 1:**
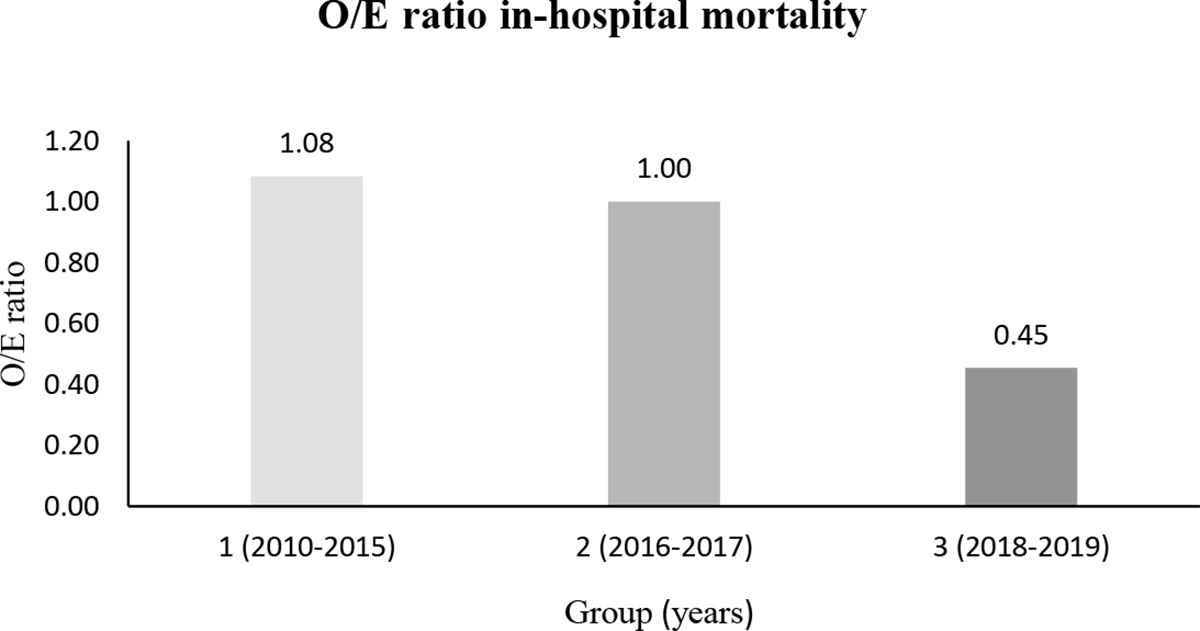
Development of observed-to-expected (O/E) ratio of in-hospital mortality after TAVI. Observed = In-hospital mortality. Expected = EuroScore II.

In-hospital outcomes are depicted in Table 3. Rates of In-hospital mortality showed a significant decrease during the observation period (9.1 % vs. 5.8 % vs. 2.5 %, p = 0.029) with a disproportionate decline of the risk-adjusted mortality (observed-to-expected (O/E) mortality: 1.08 vs. 1.00 vs. 0.45; Figure 1). Overall bleeding complications showed a downward trend including significantly lower rates of minor bleedings (8.3 % vs. 4.5 % vs. 2.5 %, p = 0.047) in group 3. The prevalence of acute kidney injury (AKIN) (all stages) sank between group 1 and 3 (53.8 % vs. 34.2 % vs. 29.2 %, p < 0.001). Furthermore, vascular access site and access-related complications decreased numerically without reaching statistical significance. Conduction disturbances including higher rates of new AV block degree 3 and new permanent pacemaker implantations were more common in group 3. Rates of hemodynamic relevant arrhythmias were significantly lower in group 3. Both length of hospitalization and stay at intensive care unit (ICU) and intermediate care unit (IMC) could be reduced significantly between group 1 and 3.

### 3.6 Predictors of in-hospital Mortality

Variables that were statistically significant in univariate analysis (Supplementary Table 6) were entered into multivariate logistic regression analysis according to their clinical relevance. Following independent predictors of in-hospital mortality could be revealed (Table 4): Age (OR: 1.103; 95 % CI: 1.013 – 1.202; p = 0.025), creatinine level before TAVI (OR: 1.497; 95 % CI: 1.013 – 2.212; p = 0.043), atrial fibrillation (OR: 2.956; 95 % CI: 1.127 – 7.749; p= 0.028) and procedure duration (OR: 1.017; 95 % CI: 1.009 – 1.025; p < 0.001).

## 4. Discussion

In this single-center analysis of 489 consecutive patients who underwent TAVI procedures at the Heart-Thorax Center Fulda from January 2010 to December 2019, we compared in-hospital outcomes between three cohorts according to ESC guideline adjustments, infrastructural, procedural and technical developments. The primary study findings were as follows: (1) The number of TAVI procedures performed annually has increased significantly; (2) Changes in patient characteristics with a clear shift from high-risk towards lower surgical risk and less comorbidities could be observed; (3) Procedural characteristics including procedure duration and contrast medium consumption could be reduced significantly; (4) Postprocedural complications decreased overall with a remarkable disproportionate decline in risk-adjusted mortality (observed-to-expected mortality); (5) The length of hospitalization as well as the stay at intensive care unit (ICU) and intermediate care unit (IMC) after TAVI could be reduced significantly.

### 4.1 In-hospital Mortality

Our results demonstrated that in-hospital mortality rates were declining significantly from 2010 to 2019 (9.1 % vs. 5.8 % vs. 2.5 %, p = 0.029). These results are substantially lower than mortality rates reported in a previous study by Akinseye et al. (5.0 %) and comparable with recently published data by the German Institute for Quality Assurance and Transparency in Healthcare (IQTIG), who reported a hospital mortality rate of 2.3 % for Germany in 2020 [9,10]. Especially the observed-to-expected mortality ratio (O/E ratio) showed a disproportionate decline with an O/E ratio of 0.45 in group 3 (years 2018–2019), which indicates that this favorable development was not only driven by selecting patients at lower surgical risk with less comorbidities, but also by improvements in procedure planning, catheter material and technique.

#### 4.1.1 Patient-related Risk Factors:

As far as patient-related factors are concerned, a clear shift from patients at high surgical risk to lower surgical risk could be observed. This is primarily due to changing indications in the European guidelines over time. Referring to the first European guideline on the management of valvular heart disease from 2007, it must be stated that TAVI was not suggested as an alternative to surgery due to limited data [6]. The first recommendation in favor of TAVI was adopted in the ESC guideline from 2012 for patients with severe symptomatic aortic stenosis who were deemed unsuitable for surgery after an individual assessment by the heart team [11]. The introduction of the ESC guideline in 2017 further specified the indications for patients with a symptomatic aortic stenosis recommending TAVI for patients ≥ 75 years, STS/ EuroSCORE II ≥ 4 % or logistic EuroSCORE I ≥10 % and further risk factors, which are not included in the risk calculators [5]. In this regard, we could observe that patients in the initial period of this study were not only older with a higher calculated surgical risk, but also had higher rates of pre-existing comorbidities, which may have influenced the mortality rates. Apart from age our study revealed renal function at baseline as a further patient-related independent predictor of in-hospital mortality. The role of renal function has already been underlined in previously used scores, such as the ACEF score (age, creatinine, ejection fraction), which was developed and validated for patients undergoing cardiac surgery [12]. Although the systolic left ventricular ejection fraction was not an independent predictor of in-hospital mortality in our multivariate analysis, we assume that a further simplification of conventional risk predicting scores by using less variables might be helpful in clinical routine. Atrial fibrillation was another patient-related predictor of hospital mortality that could be identified in multivariate regression analysis. Although the underlying mechanism for in-hospital mortality remains unclear in this study, the relevance of this comorbidity is indisputable, as there is evidence that patients with atrial fibrillation are at higher risk of rehospitalization due to heart failure after TAVI [13]. Given the fact that there was no follow-up of the patients in this study, we cannot provide further information about rehospitalizations due to heart failure.

#### 4.1.2 Infrastructural and Procedural Risk Factors:

As far as infrastructural developments are concerned, it can be stated that the hybrid operating room at our heart center was launched in 2016. Although prior studies showed no significant difference regarding midterm mortality rates in patients who underwent TAVI in hybrid operating rooms versus cardiac catheterization laboratories, we assume that patients treated in hybrid operating rooms may have benefited from various predefined logistic standards [14,15]. Technical equipment and complementary imaging methods allow instantaneous interventions in case of severe complications avoiding any considerable delay. In this context, our data could emphasize the prognostic relevance of procedure duration itself, as it was identified as an independent predictor of hospital mortality. This implies that the implementation of the hybrid operating room with all its advantages led to shorter procedure durations, which may have positively affected our hospital mortality rates. Another infrastructural aspect that needs to be discussed in this regard is the team of operators, which showed a high consistency during the observation period, apart from one cardiologist joining the team in 2016. This is directly in line with previous findings that were reported by Salemi et al. [16]. They proved that there is an inverse relationship between personal experience and the composite endpoint of in-hospital mortality, stroke and/or myocardial infarction [16]. From this it can be deduced that a constant composition of the TAVI team leads to continually growing individual and team experience, which might have had a positive impact on outcome, although procedural hospital volume itself does not play a role for outcome [17].

### 4.2 Adverse Events

Vascular complications, which are commonly associated with bleeding complications, have a major influence on mortality [18,19]. Contrary to the findings of the PARTNER study we found lower rates of major (4.7 %) and minor (8.2 %) vascular complications with a decreasing trend between group 1 (years 2010–2015) and group 3 (years 2018–2019) [18]. Several factors are known to play a role in determining vascular complications. The development of valve profiles and the use of smaller sheath sizes may have played the most substantial role in reducing vascular complications. Vascular calcification and female sex with smaller vessel diameters were described as further risk factors in previous studies [20]. However, the implementation of the planning software “3mensio” since 2016 as a mandatory part of the preprocedural planning along with advanced valve and catheter material were contributing factors for safer procedures with a lower prevalence of vascular complications in the last observation period. As far as bleeding complications are concerned, we report a slight downward trend for life-threatening and major bleedings between group 1 (years 2010–2015) and group 3 (2018–2019), while minor bleeding complications decreased significantly. There is some evidence that bleeding complications may affect the survival after TAVI [21,22]. Wang et al. could show that the occurrence of life-threatening and major bleedings after TAVI was linked to higher rates of 30-days mortality [23]. Another relevant finding of our study was the significant decrease of acute kidney injury rates, especially for stage 1 (AKIN 1) and stage 3 (AKIN 3). The association between acute kidney injury after interventional and surgical procedures and mortality has been described in numerous studies [24–27]. The reported incidence of AKIN after TAVI is heterogenous varying between 3.4 % and 57 %, which is comparable with our local results [28–30]. The etiology of an acute kidney injury following TAVI is multifactorial. Peripheral vascular disease could be identified as an important predictor for AKIN [31]. A possible explanation is that numerous procedural steps, including vascular puncture, catheter passage through an atherosclerotic vascular system or valve deployment, may increase the risk of generating emboli from atherosclerotic plaques and thus effecting the renal perfusion with a subsequent impairment of renal function. However, our analysis could show that the number of patients with peripheral vascular disease decreased over time, which might explain the declining rates of AKIN during this period. In addition, our results impressively demonstrated a significant decrease of contrast medium consumption between group 1 and 3. As far as procedure-related predictors are concerned blood transfusion has been identified as another risk factor for AKIN in several studies [32,33]. Our findings are directly in line with these studies showing a significant decrease of blood transfusions during the observed period, which can be explained by a decrease of overall bleeding complications by nearly 50 %. New permanent pacemaker (PPM) implantations due to conductance disturbances are among the most frequent complications after TAVI [34]. The rates of PPM implantations after TAVI range from 9 % to 26 % and are comparable with our local prevalence (22.8 %) [35–38]. Numerous risk factors have been discussed in previous studies. However, transcatheter heart valves still play an eminent role, although newer generation transcatheter heart valves have undergone an impressive technical progress with respect to material and valve profiles. Erkapic et al. demonstrated that self-expandable valves compared to balloon-expandable valves are associated with a higher risk of PPM implantation after TAVI [39]. In accordance with findings reported by Erkapic et al., our results showed a significant increase in PPM implantation rates, while numbers of self-expandable valve implantations raised accordingly. Nevertheless, we assume that the risk of new conductance disturbances can be minimized by comprehensive preprocedural planning strategies. In this context, a novel strategy has been introduced by Tang et al. in 2018. The so-called “cusp overlap technique” was developed to facilitate fluoroscopy-guided implantation of self-expandable transcatheter heart valves. However, this technique was introduced at the end of our study period and therefore was not an integral part of our preprocedural planning strategies at that time, which might be one explanation for the high PPI rates.

## 5. Limitations

Our analysis has some limitations. Firstly, this was a single-center retrospective study with its natural intrinsic limitations, such as selection bias or unknown confounding factors. Secondly, our study was limited by its small sample size. Thirdly, no follow-up was done as the study was designed to only assess in-hospital outcomes. Lastly, due to various developments running in parallel during the observation period, the decline in postprocedural complications needs to be considered as an overall result of the interplay between changing guideline indications, technical, procedural and institutional developments.

## 6. Conclusion

In this investigation, the aim was to assess determinants that have affected in-hospital outcomes after TAVI. Our study has shown that the patient-related characteristics age, creatinine level before TAVI, the presence of atrial fibrillation and procedure duration are independent predictors for in-hospital mortality. Although these predictors decreased along with other comorbidities during the observation period, the decline in hospital-mortality was disproportionate, which was indicated by an observed-to-expected mortality ratio of 0.45 for the third treatment period. From this, it can be assumed that apart from patient-related risk factors, there were further institutional, technical and procedural developments, which ran in parallel and had a major impact on postprocedural outcomes, especially on hospital mortality rates after TAVI.

## Supplementary Material

1

## Figures and Tables

**Table 1:** Patient characteristics: Demography and cardiovascular risk factors.

	All (2010–2019) n = 489	Group 1 (2010–2015) n = 132	Group 2 (2016–2017) n = 155	Group 3 (2018–2019) n = 202	p value

**Demographic characteristics**					

**Age (years)**					

- Mean (± SD)	81.8 (± 5.4)	82.1 (± 6.2)	82.5 (± 4.8)	81.1 (± 5.1)	**0.012**

- Median (IQR)	82.0 (6.0)	83.0 (8.0)	83.0 (6.0)	81.0 (5.0)	

**Male - n (%)**	239 (48.9 %)	62 (47.0 %)	68 (43.9 %)	109 (54.0 %)	0.147

**BMI (kg/m^2^)**					

- Mean (± SD)	27.7 (± 5.1)	26.9 (± 4.4)	27.6 (± 5.8)	28.3 (± 4.9)	**0.030**

- Median (IQR)	27.1 (5.9)	27.0 (5.7)	26.7 (5.0)	27.6 (6.8)	

**BSA (m^2^)**					

- Mean (± SD)	1.8 (± 0.2)	1.8 (± 0.2)	1.8 (± 0.2)	1.9 (± 0.2)	**0.002**

- Median (IQR)	1.8 (0.3)	1.8 (0.2)	1.8 (0.2)	1.9 (0.3)	

**Logistic** **EuroSCORE (%)**					

- Mean (± SD)	20.2 (± 14.2)	26.7 (± 14.8)	18.3 (± 12.8)	17.4 (± 13.6)	**< 0.001**

- Median (IQR)	15.7 (16.5)	24 (19.4)	14.4 (13.3)	12.7 (10.9)	

**EuroSCORE II (%)**					

- Mean (± SD)	6.4 (± 5.4)	8.4 (± 6.0)	5.8 (± 4.9)	5.5 (± 5.0)	**< 0.001**

- Median (IQR)	4.7 (5.3)	6.6 (6.5)	4.4 (4.6)	3.7 (3.6)	

**Cardiovascular** **Risk factors**					

**Hypertension - n (%)**	452 (92.4 %)	124 (93.9 %)	145 (93.5 %)	183 (90.6 %)	0.432

**Diabetes mellitus - n (%)**	171 (35.0 %)	52 (39.4 %)	46 (29.7 %)	73 (36.1 %)	0.205

**Hyperlipidemia - n (%)**	287 (58.7 %)	82 (62.1 %)	91 (58.7 %)	114 (56.4 %)	0.587

**Renal insufficiency - n (%)**	268 (54.8 %)	81 (61.4 %)	78 (50.3 %)	109 (54.0 %)	0.165

- Dialysis - n (%)	13 (2.7 %)	6 (4.5 %)	1 (0.6 %)	6 (3.0 %)	0.115

**Obesity**					**0.006**
- Obesity grade 1	94 (19.2 %)	25 (18.9 %)	22 (14.2 %)	47 (23.3 %)	
- Obesity grade 2	30 (6.1 %)	3 (2.3 %)	7 (4.5 %)	20 (9.9 %)	
- Obesity grade 3	6 (1.2 %)	1 (0.8 %)	4 (2.6 %)	1 (0.5 %)	

**Smoking - n (%)**	25 (5.1 %)	2 (1.5 %)	8 (5.2 %)	15 (7.4 %)	0.056

**Family history of cardiovascular disease – n (%)**	110 (22.5 %)	36 (27.3 %)	29 (18.7 %)	45 (22.3 %)	0.222

Abbreviations: BMI = Body Mass Index; BSA = Body Surface Area; IQR = interquartile range; SD = standard deviation.

**Table 2:** Patient characteristics: Clinical features and comorbidities.

	All (2010–2019) n = 489	Group 1 (2010–2015) n = 132	Group 2 (2016–2017) n = 155	Group 3 (2018–2019) n = 202	p value
**Clinical features**					

**NYHA class**					0.164

NYHA I - n (%)	2 (0.4 %)	2 (1.5 %)	0 (0.0 %)	0 (0.0 %)	

NYHA II - n (%)	61 (12.5 %)	17 (12.9 %)	22 (14.2 %)	22 (10.9 %)	

NYHA III - n (%)	370 (75.7 %)	94 (71.2 %)	114 (73.5 %)	162 (80.2 %)	

NYHA IV - n (%)	52 (10.6 %)	17 (12.9 %)	19 (12.3 %)	16 (7.9 %)	

**CCS class**					**< 0.001**

CCS I - n (%)	15 (3.1 %)	3 (2.3 %)	1 (0.6 %)	11 (5.4 %)	

CCS II - n (%)	233 (47.6 %)	34 (25.8 %)	114 (73.5 %)	85 (42.1 %)	

CCS III - n (%)	62 (12.7 %)	47 (35.6 %)	10 (6.5 %)	5 (2.5 %)	

CCS IV - n (%)	1 (0.2 %)	1 (0.8 %)	0 (0.0 %)	0 (0.0 %)	

**Cardiac decompensation - n (%)**	177 (36.2 %)	60 (45.5 %)	54 (34.8 %)	63 (31.2 %)	**0.027**

**Comorbidities**					

**Coronary artery disease - n (%)**					0.433

- 1-vessel disease - n (%)	100 (20.4 %)	27 (20.5 %)	31 (20.0 %)	42 (20.8 %)	
- 2-vessel disease - n (%)	70 (14.3 %)	15 (11.4 %)	23 (14.8 %)	32 (15.8 %)	
- 3-vessel disease - n (%)	112 (22.9 %)	38 (28.8 %)	29 (18.7 %)	45 (22.3 %)	
- left main artery stenosis - n (%)	21 (4.3 %)	8 (6.1 %)	9 (5.8 %)	4 (2.0 %)	

**Previous myocardial infarction**					**0.018**

- no myocardial infarction	68 (13.9 %)	28 (21.2 %)	17 (11.0 %)	23 (11.4 %)	

- STEMI – n (%)	32 (6.5 %)	17 (12.9 %)	6 (3.9 %)	9 (4.5 %)	

- NSTEMI – n (%)	34 (7.0 %)	13 (9.8 %)	10 (6.5 %)	11 (5.4 %)	

**Previous bypass operation- n (%)**	79 (16.2 %)	33 (25.0 %)	17 (11.0 %)	29 (14.4 %)	**0.004**

**Previous valve operation - n (%)**	14 (2.9 %)	4 (3.0 %)	3 (1.9 %)	7 (3.5 %)	0.685

**Previous balloon valvuloplasty - n (%)**	37 (7.6 %)	1 (0.8 %)	16 (10.3 %)	20 (9.9 %)	**0.002**

**Previous PCI - n (%)**	199 (40.7 %)	57 (43.2 %)	60 (38.7 %)	82 (40.6 %)	0.744

**PVD - n (%)**	118 (24.1 %)	51 (38.6 %)	37 (23.9 %)	30 (14.9 %)	**< 0.001**

**CVD - n (%)**	62 (12.7 %)	30 (22.7 %)	15 (9.7 %)	17 (8.4 %)	**< 0.001**

**COPD - n (%)**	57 (11.7 %)	18 (13.6 %)	14 (9.0 %)	25 (12.4 %)	0.440

**Previous stroke - n (%)**	51 (10.4 %)	19 (14.4 %)	15 (9.7 %)	17 (8.4 %)	0.203

**History of cancer - n (%)**	123 (25.2 %)	28 (21.2 %)	51 (32.9 %)	44 (21.8 %)	**0.027**

**Anemia – n (%)**	230 (47.1 %)	77 (58.3 %)	67 (43.5 %)	86 (42.6 %)	**0.010**

Abbreviations: CCS = Canadian Cardiovascular Society; COPD = chronic obstructive pulmonary disease; CVD = cerebrovascular disease; NSTEMI = Non-ST-elevation myocardial infarction; NYHA = New York Heart Association; PCI = percutaneous coronary intervention; PVD = peripheral vascular disease; STEMI = ST-elevation myocardial infarction.

**Table 3:** Postprocedural outcomes.

	All(2010–2019)n = 489	Group 1 (2010–2015) n = 132	Group 2 (2016–2017) n = 155	Group 3 (2018–2019) n = 202	p value
**Postprocedural outcomes**					
**In-hospital mortality - n (%)**	26 (5.3 %)	12 (9.1 %)	9 (5.8 %)	5 (2.5 %)	**0.029**
**Myocardial infarction - n (%)**	2 (0.4 %)	2 (1.5 %)	0 (0.0 %)	0 (0.0 %)	0.066
**Neurological complications (all) - n (%)**	11 (2.2 %)	2 (1.5 %)	3 (1.9 %)	6 (3.0 %)	0.647
- Stroke - n (%)	9 (1.8 %)	2 (1.5 %)	2 (1.3 %)	5 (2.5 %)	0.675
- TIA - n (%)	2 (0.4 %)	0 (0.0 %)	1 (0.6 %)	1 (0.5 %)	0.673
**Bleeding complications (all) - n (%)**	53 (10.8 %)	19 (14.4 %)	18 (11.6 %)	16 (7.9 %)	0.165
- Life-threatening bleeding - n (%)	18 (3.7 %)	5 (3.8 %)	6 (3.9 %)	7 (3.5 %)	0.977
- Major bleeding - n (%)	12 (2.5 %)	3 (2.3 %)	5 (3.2 %)	4 (2.0 %)	0.743
- Minor bleeding - n (%)	23 (4.7 %)	11 (8.3 %)	7 (4.5 %)	5 (2.5 %)	**0.047**
- Blood transfusion - n (%)	121 (24.7 %)	47 (35.6 %)	40 (25.8 %)	34 (16.8 %)	**< 0.001**
- Transfusion of thrombocyte concentrates - n (%)	21 (4.3 %)	7 (5.3 %)	7 (4.5 %)	7 (3.5 %)	0.711
- Transfusion of fresh frozen plasma - n (%)	20 (4.1 %)	7 (5.3 %)	8 (5.2 %)	5 (2.5 %)	0.318
**Acute Kidney Injury (AKIN) (all) - n (%)**	183 (37.4 %)	71 (53.8 %)	53 (34.2 %)	59 (29.2 %)	**< 0.001**
- AKIN I - n (%)	152 (31.1 %)	57 (43.2 %)	48 (31.0 %)	47 (23.3 %)	**0.001**
- AKIN II - n (%)	3 (0.6 %)	0 (0.0 %)	0 (0.0 %)	3 (1.5 %)	0.117
- AKIN III - n (%)	28 (5.7 %)	14 (10.6 %)	5 (3.2 %)	9 (4.5 %)	**0.016**
- Dialysis after TAVI (all) - n (%)	24 (4.9 %)	11 (8.3 %)	4 (2.6 %)	9 (4.5 %)	0.074
- Acute dialysis after TAVI - n (%)	13 (2.7 %)	6 (4.5 %)	4 (2.6 %)	3 (1.5 %)	0.235
**Vascular complications (all) – n (%)**	66 (13.5 %)	20 (15.2 %)	20 (12.9 %)	26 (12.9 %)	0.809
- Major vascular complication - n (%)	23 (4.7 %)	7 (5.3 %)	6 (3.9 %)	10 (5.0 %)	0.83
- Minor vascular complication - n (%)	40 (8.2 %)	12 (9.1 %)	12 (7.7 %)	16 (7.9 %)	0.903
- Percutaneous closure device failure- n (%)	4 (0.8 %)	1 (0.8 %)	2 (1.3 %)	1 (0.5 %)	0.708
**Conduction disturbances andarrhythmias (all) - n (%)**	242 (49.5 %)	64 (48.5 %)	77 (49.7 %)	101 (50.0 %)	0.962
- New AV block 1 - n (%)	48 (9.8 %)	11 (8.3 %)	16 (10.3 %)	21 (10.4 %)	0.799
- New AV block 2 - n (%)	7 (1.4 %)	1 (0.8 %)	2 (1.3 %)	4 (2.0 %)	0.645
- New AV block 3 - n (%)	35 (7.2 %)	5 (3.8 %)	6 (3.9 %)	24 (11.9 %)	**0.003**
- New LBBB - n (%)	109 (22.3 %)	24 (18.2 %)	36 (23.2 %)	49 (24.3 %)	0.403
- New RBBB - n (%)	6 (1.2 %)	2 (1.5 %)	1 (0.6 %)	3 (1.5 %)	0.728
- New LAFB - n (%)	34 (7.0 %)	17 (12.9 %)	11 (7.1 %)	6 (3.0 %)	**0.002**
- New onset atrial fibrillation - n (%)	24 (4.9 %)	4 (3.0 %)	7 (4.5 %)	13 (6.4 %)	0.357
- Hemodynamic relevant arrhythmia-n (%)	29 (5.9 %)	6 (4.5 %)	16 (10.3 %)	7 (3.5 %)	**0.018**
- New permanent pacemaker implantation - n (%)	73 (14.9 %)	10 (7.6 %)	17 (11.0 %)	46 (22.8 %)	**< 0.001**
**Valve malposition (all) - n (%)**	5 (1.0 %)	0 (0.0 %)	3 (1.9 %)	2 (1.0 %)	0.267
- Valve migration - n (%)	1 (0.2 %)	0 (0.0 %)	1 (0.6 %)	0 (0.0 %)	0.34
- Valve embolization - n (%)	4 (0.8 %)	0 (0.0 %)	2 (1.3 %)	2 (1.0 %)	0.452
- Ectopic valve deployment - n (%)	0 (0.0 %)	0 (0.0 %)	0 (0.0 %)	0 (0.0 %)	
**Other Complications**					
- Ventricle injury - n (%)	6 (1.2 %)	2 (1.5 %)	2 (1.3 %)	2 (1.0 %)	0.909
- Pericardial tamponade - n (%)	8 (1.6 %)	2 (1.5 %)	4 (2.6 %)	2 (1.0 %)	0.492
- Sepsis - n (%)	5 (1.0 %)	3 (2.3 %)	1 (0.7 %)	1 (0.5 %)	0.252
- Endocarditis - n (%)	1 (0.2 %)	0 (0.0 %)	1 (0.7 %)	0 (0.0 %)	0.328
**Paravalvular insufficiency - n (%)**					0.401
- Grade 1	127 (26.0 %)	30 (22.7 %)	38 (24.5 %)	59 (29.2 %)	
- Grade 2	23 (4.7 %)	4 (3.0 %)	7 (4.5 %)	12 (5.9 %)	
- Grade 3	0 (0.0 %)	0 (0.0 %)	0 (0.0 %)	0 (0.0 %)	
**Hospitalization after TAVI (days)**					
- Mean (± SD)	11.1 (± 10.2)	11.8 (± 6.4)	11.5 (± 14.2)	10.4 (± 8.4)	**< 0.001**
- Median (IQR)	8.0 (6.0)	9.0 (8.0)	8.0 (6.0)	8.0 (6.0)	
**Stay at ICU und IMC after TAVI (days)**					
- Mean (± SD)	4.4 (± 6.1)	4.8 (± 5.0)	4.6 (± 4.1)	2.6 (± 7.1)	**0.001**
- Median (IQR)	3.0 (3.0)	3.0 (5.0)	3.0 (4.0)	2.0 (2.0)	

Abbreviations: AV = Atrioventricular; ICU = Intensive Care Unit; IMC = Intermediate Care Unit; IQR = Interquartile Range; LAFB = Left Anterior Fascicular Block; LBBB = Left Bundle Branch Block; RBBB = Right Bundle Branch Block; SD = Standard Deviation; TIA = Transient Ischemic Attack.

**Table 4:** Multivariate analysis: In-hospital mortality.

Multivariate analysis: In-hospital mortality			
Variables	p value	Odds Ratio (OR)	95 % Confidence Interval (CI)
Group	0.405	1.723	0.479 – 6.197
Age (years)	**0.025**	1.103	1.013 – 1.202
Creatinine before TAVI (mg/dl)	**0.043**	1.497	1.013 – 2.212
Atrial fibrillation before TAVI	**0.028**	2.956	1.127 – 7.749
PVD	0.101	2.284	0.851 – 6.134
CT scan before TAVI	0.527	0.631	0.151 – 2.632
Hybrid OR	0.703	0.732	0.148 – 3.625
Edwards THV	0.523	0.656	0.180 – 2.394
Procedure duration (min)	**< 0.001**	1.017	1.009 – 1.025
			
**R^2^**	0.269		
**Significance (Chi^2^)**	**< 0.001**		

Abbreviations: CT = Computer Tomography; OR = Operating Room; PVD = Peripheral Vascular Disease; R2 = Nagelkerke R Square; TAVI = Transcatheter Aortic Valve Implantation; THV = Transcatheter Heart Valve.

## Data Availability

The dataset can be provided by the corresponding author on demand.
