# Supplementary material for: Development of In-Hospital Outcomes in Patients undergoing Transcatheter Aortic Valve Implantation (TAVI) at an Interdisciplinary Heart Center: A Single-Center Experience of 489 Consecutive Cases
Source: Cardiol Cardiovasc Med. Author manuscript; Available in PMC 2023 May 9. (PMC10167775; doi:10.26502/fccm.92920309)
Supplement: 1 [file NIHMS1883380-supplement-1.pdf]

Supplementary Data

Supplementary Table 1: Blood examination before TAVI. Abbreviations: IQR = Interquartile Range; SD = Standard Deviation.

	**All (2010 - 2019) n = 489**	**Group 1 (2010 – 2015)n = 132**	**Group 2 (2016 – 2017)n = 155**	**Group 3 (2018 – 2019) n = 202**	**p value**
**Blood examination**					
**Creatinine (mg/dl)**					
- Mean (± SD)	1.4 (± 0.9)	1.6 (± 1.1)	1.2 (± 0.4)	1.3 (± 0.9)	**0.005**
- Median (IQR)	1.2 (0.6)	1.3 (0.8)	1.2 (0.5)	1.1 (0.5)	
**NT-pro-BNP (pg/ml)**					
- Mean (± SD)	3483.7(± 5217.4)	4928,1(± 6792.7)	3259.8(± 5202.9)	2769.1(± 3807.1)	< **0.001**
- Median (IQR)	1636 (2845.0)	2535,5 (4725.0)	1409 (2482.0)	1262 (2416.0)	

Comment [MR17]: name of every table must be fat.

Comment [MR18]: please make sure that these headings are in the right position in the final version. Unfortunately, these names were standing in the middle of the column in the last version. These names must be positionned at the left side of the column!

**Troponin-T (pg/ml)**					
- Mean (± SD)	42.3 (± 124.6)	64.5 (± 223.4)	38.0 (± 95.0)	33.3 (± 34.5)	0.217
- Median (IQR)	25.0 (23.0)	30.5 (29.0)	24.0 (18.0)	25.0 (22.0)	
**Hemoglobin (g/dl)**					
- Mean (± SD)	12.5 (± 1.8)	12.1 (± 1.9)	12.5 (± 1.7)	12.6 (± 1.8)	**0.009**
- Median (IQR)	12.6 (± 2.2)	12.1 (2.4)	12.7 (2.2)	12.9 (2.3)	
**Platelets (Thousand/µl)**					
- Mean (± SD)	214.7 (± 82.9)	206.9 (± 85.1)	218.7 (± 97.1)	216.8 (± 68.7)	0.157
- Median (IQR)	202.0 (82.0)	198.5 (83.0)	201.0 (75.0)	208.0 (90.0)	

Supplementary Table 2: **Echocardiographic measurements before TAVI.** Abbreviations: AVA = Aortic Valve Area; IQR = Interquartile Range; LV = Left Ventricular; LVEF = Left Ventricular Ejection Fraction; MPG = Mean Pressure Gradient.

	**All (2010-2019) n = 489**	**Group 1 (2010-2015) n = 132**	**Group 2 (2016-2017) n = 155**	**Group 3 (2018-2019) n = 202**	**p value**
**Echocardiography**					
**LVEF (%)**					
- Mean (± SD)	52.0 (± 10.1)	49.7 (± 9.7)	53.8 (± 9.8)	52.2 (± 10.2)	**< 0.001**
- Median (IQR)	55.0 (5.0)	55.0 (10.0)	55.0 (0.0)	55.0 (5.0)	
**LV function**					**0.005**
- Normal systolic LV function – n (%)	382 (78.1 %)	90 (68.2 %)	132 (85.2 %)	160 (79.2 %)	
- Moderately reduced LV function – n (%)	30 (6.1 %)	15 (11.4 %)	4 (2.6 %)	11 (5.4 %)	
- Severely reduced LV function – n (%)	77 (15.7 %)	27 (20.5 %)	19 (12.3 %)	31 (15.3 %)	
**AVA (cm^2)**					

Comment [MR19]: The name of a table should be fat.

Comment [MR20]: fat!

- Mean (± SD)	0.74 (± 0.16)	0.70 (± 0.14)	0.72 (± 0.16)	0.78 (± 0.15)	**0.001**
- Median (IQR)	0.74 (0,23)	0.70 (0.20)	0.70 (0.27)	0.80 (0.21)	
**MPG (mmHg)**					
- Mean (± SD)	42.2 (± 13.8)	39.6 (± 15.0)	43.4 (± 14.0)	43.0 (± 12.6)	0.051
- Median (IQR)	42.0 (16.0)	40.0 (21.0)	43.0 (14.0)	43.0 (13.5)	
**Aortic annulus diameter (mm)**					
- Mean (± SD)	23.4 (± 3.2)	23.6 (± 2.1)	22.7 (± 3.4)	24.1 (4.5)	0.039
- Median (IQR)	23.0 (4.0)	23.3 (3.0)	22.0 (3.6)	23.5 (5.3)	

Supplementary Table 3: **Computer Tomography before TAVI.** Abbreviations: CT = Computer Tomography; IQR = Interquartile Range; LCA = Left Coronary Artery; RCA = Right Coronary Artery; SD = Standard Deviation.

Comment [MR21]: name of a table must be fat.

	**All (2010-2019) n = 456**	**Group 1 (2010-2015) n = 100**	**Group 2 (2016-2017) n = 154**	**Group 3 (2018-2019) n = 202**	**p value**
**Computer Tomography (CT)**					
CT scan prior to TAVI	456 (93.3 %)	100 (75.8 %)	154 (99.4 %)	202 (100 %)	< **0.001**
**Aortic annulus diameter (mm)**					
- Mean (± SD)	24.7 (± 2.6)	24.5 (± 3.8)	24.4 (± 2.2)	24.9 (± 2.2)	0.219
- Median (IQR)	24.6 (3.3)	24.5 (3.0)	24.3 (3.4)	24.8 (3.2)	
**Area derived diameter (mm)**					
- Mean (± SD)	24.6 (± 2.2)	---	24.2 (± 2.1)	24.8 (± 2.2)	0.051
- Median (IQR)	24.4 (3.5)	---	24.1 (3.5)	24.5 (3.3)	
**Perimeter derived diameter(mm)**					
- Mean (± SD)	24.9 (± 2.2)	---	24.6 (± 2.2)	25.2 (± 2.2)	**0.039**

Comment [MR22]: space was added between „Tomography“ and „(CT)“

- Median (IQR)	24.9 (3.4)	---	24.5 (3.4)	25.0 (3.3)	
**Distance RCA to aorticannulus (mm)**					
- Mean (± SD)	1.7 (± 0.4)	1.5 (± 0.3)	1.6 (± 0.4)	1.8 (± 0.4)	< **0.001**
- Median (IQR)	1.6 (0.5)	1.5 (0.3)	1.6 (0.5)	1.8 (0.5)	
**Distance LCA to aortic annulus (mm)**					
- Mean (± SD)	1.4 (± 0.3)	1.4 (± 0.3)	1.3 (± 0.3)	1.4 (± 0.3)	**0.021**
- Median (IQR)	1.4 (0.4)	1.4 (0.4)	1.3 (0.4)	1.4 (0.4)	
**Minimal diameter right femoral artery (mm)**					
- Mean (± SD)	7.3 (± 1.5)	7.7 (± 1.6)	7.4 (± 1.2)	7.0 (± 1.6)	**0.006**
- Median (IQR)	7.1 (1.7)	8.0 (2.0)	7.3 (1.5)	7.0 (2.0)	
**Minimal diameter right external iliac artery (mm)**					
- Mean (± SD)	7.2 (± 1.7)	7.6 (± 1.3)	7.2 (± 2.1)	7.0 (± 1.5)	**0.002**
- Median (IQR)	7.0 (1.8)	8.0 (1.0)	7.0 (1.9)	6.9 (1.9)	
**Minimal diameter left external iliac artery (mm)**					
- Mean (± SD)	7.3 (± 1.5)	7.7 (± 1.3)	7.2 (± 1.7)	7.1 (± 1.4)	**0.004**
- Median (IQR)	7.2 (1.8)	8.0 (1.0)	7.0 (2.0)	7.1 (1.9)	
**Kinking – n (%)**	65 (13.3 %)	29 (22.0 %)	20 (12.9 %)	16 (7.9 %)	**0.001**
**Porcelain aorta – n (%)**	12 (2.5 %)	10 (7.6 %)	2 (1.3 %)	0 (0.0 %)	< **0.001**
**Aortic aneurysm - n (%)**	18 (3.7 %)	6 (4.5 %)	7 (4.5 %)	5 (2.5 %)	0.494

Supplementary Table 4: Electrocardiography (ECG) before TAVI. Abbreviations: AV = Atrioventricular; LAFB = Left Anterior Fascicular Block; LBBB = Left Bundle Branch Block; RBBB = Right Bundle Branch Block.

Comment [MR23]: fat!

Comment [MR24]: Name of the table must be fat.

Comment [MR25]: make sure that this heading is at the right position in the final version. Unfortunately, it was not in the last version. It must be at the left side. Furthermore, space was added between „ Electrocardiography“ and „(ECG)“

	All (2010-2019) n = 489	Group 1 (2010-2015) n = 132	Group 2 (2016-2017) n = 155	Group 3 (2018-2019) n = 202	p value
**Electrocardiography (ECG)**					
Atrial fibrillation – n (%)	146 (29.9 %)	50 (37.9 %)	42 (27.3 %)	54 (26.7 %)	0.065
Pacemaker – n (%)	51 (10.4 %)	14 (10.6 %)	11 (7.1 %)	26 (12.9 %)	0.208
AV block 1 - n (%)	104 (21.3 %)	32 (24.2 %)	33 (21.4 %)	39 (19.3 %)	0.559
AV block 2 - n (%)	1 (0.2 %)	1 (0.8 %)	0 (0.0 %)	0 (0.0 %)	0.259
AV block 3 - n (%)	2 (0.4 %)	0 (0.0 %)	2 (1.3 %)	0 (0.0 %)	0.113
LBBB - n (%)	40 (8.2 %)	15 (11.4 %)	8 (5.2 %)	17 (8.4 %)	0.164
RBBB - n (%)	59 (12.1 %)	20 (15.2 %)	16 (10.4 %)	23 (11.4 %)	0.432
LAFB - n (%)	67 (13.7 %)	9 (6.8 %)	27 (17.5 %)	31 (15.3 %)	**0.022**

Supplementary Table 5: **Procedural characteristics.** Abbreviations: F = French; IQR = Interquartile Range; SD = Standard Deviation; THV = Transcatheter Heart Valve.

Comment [MR26]: Name of the table must be fat.

	All (2010-2019) n = 489	Group 1 (2010-2015) n = 132	Group 2 (2016-2017) n = 155	Group 3 (2018-2019) n = 202	p value
**General procedural characteristics**					
**Procedure duration (min)**					
- Mean (± SD)	81.2 (± 49.1)	112.7 (± 46.9)	81.5 (± 48.2)	60.4 (± 39.3)	< **0.001**
- Median (IQR)	71.0 (43.0)	97.0 (52.0)	71.5 (36.0)	54.5 (31.0)	
**Contrast medium (ml)**					
- Mean (± SD)	139.5 (± 72.1)	136.0 (± 66.7)	167.6 (± 84.6)	119.6 (± 56.7)	< **0.001**
- Median (IQR)	124.0 (95.0)	123.5 (100.0)	147.0 (105.0)	110.0 (70.0)	
**Fluoroscopy time (min)**					

- Mean (± SD)	10.6 (± 6.0)	9.6 (± 6.8)	10.8 (± 5.8)	11.0 (± 5.4)	**0.003**
- Median (IQR)	9.7 (8.2)	7.1 (10.9)	9.7 (7.0)	10.0 (7.2)	
**Radiation dose (cGycm^2)**					
- Mean (± SD)	2900.1(± 1949.7)	2728.7 (± 1595.0)	2715.2(± 1906.3)	3172.4(± 2178.7)	0.056
- Median (IQR)	2463.5 (2013)	2470 (1768)	2235.5 (1530)	2799 (2414)	
**Access route and valve types**					
Transapical	144 (29.4 %)	80 (60.6 %)	41 (26.5 %)	23 (11.4 %)	**< 0.001**
Valve types					**< 0.001**
Balloon-expandable	239 (48.9 %)	132 (100 %)	60 (38.7 %)	47 (23.3 %)	
Self-expandable	250 (51.1 %)	0 (0.0 %)	95 (61.3 %)	155 (76.7 %)	
**Valve models: Balloon-expandable THV**					
- Edwards SAPIEN	12 (2.5 %)	12 (9.1 %)	0 (0.0 %)	0 (0.0 %)	
- Edwards SAPIEN XT	79 (16.2 %)	79 (59.8 %)	0 (0.0 %)	0 (0.0 %)	
- Edwards SAPIEN 3	148 (30.3 %)	41 (31.1 %)	60 (38.7 %)	47 (23.2 %)	
**Valve models: Self-expandable THV**					
- Symetis ACURATE Neo	67 (13.7 %)	0 (0.0 %)	37 (23.9 %)	30 (14.9 %)	
- Symetis ACURATE TA	15 (3.1 %)	0 (0.0 %)	15 (9.7 %)	0 (0.0 %)	
- CoreValve Evolut R	168 (34.4 %)	0 (0.0 %)	43 (27.7 %)	125 (61.9 %)	
**Valve sizes (mm)**					**< 0.001**
- 20 mm	1 (0.2 %)	1 (0.8 %)	0 (0.0 %)	0 (0.0 %)	
- 23 mm	97 (19.8 %)	46 (34.8 %)	34 (21.9 %)	17 (8.4 %)	
- 25 mm	30 (6.1 %)	0 (0.0 %)	18 (11.6 %)	12 (5.9 %)	
- 26 mm	129 (26.4 %)	64 (48.5 %)	29 (18.7 %)	36 (17.8 %)	

Comment [MR27]: space was added.

- 27 mm	33 (6.7 %)	0 (0.0 %)	18 (11.6 %)	15 (7.4 %)	
- 29 mm	140 (28.6 %)	21 (15.9 %)	45 (29.0 %)	74 (36.6 %)	
- 34 mm	59 (12.1 %)	0 (0.0 %)	11 (7.1 %)	48 (23.8 %)	
**Sheath sizes (F)**					< **0.001**
- 14 F	232 (67.2 %)	19 (36.5 %)	91 (79.8 %)	122 (68.2 %)	
- 16 F	83 (24.1 %)	3 (5.8 %)	23 (20.2 %)	57 (31.8 %)	
- 18 F	11 (3.2 %)	11 (21.2 %)	0 (0.0 %)	0 (0.0 %)	
- 19 F	19 (5.5 %)	19 (36.5 %)	0 (0.0 %)	0 (0.0 %)	
**Specific procedural characteristics**					
Predilatation - n (%)	304 (62.2 %)	131 (99.2 %)	109 (70.3 %)	64 (31.7 %)	< **0.001**
Rapid Pacing - n (%)	351 (71.8 %)	132 (100 %)	114 (73.5 %)	105 (52.0 %)	< **0.001**
Postdilatation - n (%)	70 (14.3 %)	13 (9.8 %)	17 (11.0 %)	40 (19.8 %)	**0.014**
Conversion to surgery - n (%)	5 (1.0 %)	2 (1.5 %)	2 (1.3 %)	1 (0.5 %)	0.612
Conversion to transapical access - n (%)	6 (1.2 %)	4 (3.0 %)	2 (1.3 %)	0 (0.0 %)	**0.048**
Cardiopulmonary bypass - n (%)	9 (1.8 %)	4 (3.0 %)	4 (2.6 %)	1 (0.5 %)	0.171
Valve-in-Valve – n (%)	6 (1.2 %)	2 (1.5 %)	1 (0.6 %)	3 (1.5 %)	0.728

Comment [MR28]: space was added-> two seperate words

Supplementary Table 6: **Univariate logistic regression analysis.** Abbreviations as previously mentioned.

Comment [MR29]: Name of the table must be fat.

**Univariate logistic regression analysis: In-hospital mortality**			
**Variables**	**p value**	**Odds Ratio (OR)**	**95 % Confidence interval (CI)**
Group (years)	**0.01**	0.518	0.313 – 0.855
**Demography**			

Age (years)	**0.036**	1.095	1.006 – 1.192
Gender	0.189	0.581	0.258 – 1.307
Body mass index (kg/m^2)	**0.009**	0.868	0.781 – 0.966
Body surface area (m^2)	0.146	0.21	0.026 – 1.724
Logistic EuroSCORE (%)	**< 0.001**	1.051	1.029 – 1.073
EuroSCORE II (%)	0.057	1.057	0.998 – 1.120
**Risk factors**			
Hypertension	0.436	0.608	0.174 – 2.127
Diabetes mellitus	0.379	0.672	0.277 – 1.631
Hyperlipidemia	0.607	0.812	0.367 – 1.794
Renal insufficiency	0.061	2.333	0.962 – 5.656
Obesity	0.087	0.345	0.102 – 1.169
Family history of cardiovascular disease	0.942	1.036	0.405 – 2.646
**Clinical features**			
Cardiac decompensation	**0.023**	2.534	1.137 – 5.645
NYHA class	0.978	1.382	0.648 – 2.945
CCS class	0.627	0.916	0.642 – 1.306
**Comorbidities**			
Myocardial infarction	0.055	2.428	0.980 – 6.017
- STEMI	0.298	1.952	0.553 – 6.885
- NSTEMI	0.094	2.624	0.850 – 8.106
Coronary heart disease	0.998	1.001	0.450 – 2.227
- 1-vessel disease	0.733	1.178	0.460 – 3.015
- 2-vessels disease	0.679	0.771	0.225 – 2.639

- 3-vessels disease	0.983	1.01	0.396 – 2.581
- left main artery stenosis	0.076	3.225	0.886 – 11.739
Previous bypass operation	0.114	0.197	0.026 – 1.479
Previous balloon valvuloplasty	0.98	1.019	0.231 – 4.490
Previous valve intervention	0.149	3.132	0.663 – 14.789
Previous PCI	0.561	1.265	0.572 – 2.796
Peripheral vascular disease	**0.009**	2.887	1.296 – 6.431
Cerebrovascular disease	**0.031**	2.733	1.099 – 6.798
COPD	0.07	2.424	0.930 – 6.314
Previous stroke	0.14	2.158	0.777 – 5.996
History of cancer	0.113	0.373	0.110 – 1.264
Anemia	0.482	1.329	0.602 – 2.935
**Blood examination**			
Creatinine before TAVI (mg/dl)	**0.002**	1.498	1.161 – 1.933
Hemoglobin before TAVI (g/dl)	0.506	0.929	0.749 – 1.153
NT-pro-BNP before TAVI (pg/ml)	0.062	1	1.000 – 1.000
Troponin-T before TAVI (pg/ml)	0.944	1	0.997 – 1.003
Platelets before TAVI (Tsd. /µl)	**0.026**	0.992	0.985 – 0.999
**Echocardiography**			
LVEF (%)	0.512	0.988	0.952 – 1.025
Normal systolic LV function (≥ 50 %)	0.9	0.942	0.368 – 2.407
Moderately reduced LV function (41-49 %)	0.248	2.106	0.595 – 7.458

Comment [MR30]: space was added

Severely reduced LV function (≤ 40 %)	0.53	0.675	0.198 – 2.305
AVA (cm²)	**< 0.001**	0.007	0.000 – 0.110
MPG (mmHg)	0.177	0.979	0.949 – 1.010
Diameter aortic annulus (Echocardiography) (mm)	0.788	1.022	0.871 – 1.199
**Computer tomography (CT)**			
CT prior to TAVI	**0.002**	0.206	0.077 – 0.556
Diameter aortic annulus (mm)	0.412	1.094	0.883 – 1.355
Area-derived diameter (mm)	0.333	1.139	0.875 – 1.484
Perimeter-derived diameter (mm)	0.271	1.159	0.891 – 1.506
Distance right coronary artery to aortic annulus (mm)	0.34	0.518	0.134 – 2.000
Distance left coronary artery to aortic annulus (mm)	0.528	0.613	0.134 – 2.803
Minimal diameter right femoral artery (mm)	0.664	0.928	0.664 – 1.299
Minimal diameter right external iliac artery (mm)	0.573	0.914	0.668 – 1.250
Minimal diameter left external iliac artery (mm)	0.399	0.867	0.623 – 1.207
Kinking	0.747	1.198	0.399 – 3.596
**Electrocardiography (ECG)**			
Atrial fibrillation before TAVI	**0.037**	2.419	1.056 – 5.540
Permanent pacemaker before TAVI	0.283	0.33	0.044 – 2.491
AV block degree 1 before TAVI	0.821	1.114	0.436 – 2.850
LBBB before TAVI	0.923	0.93	0.212 – 4.085
RBBB before TAVI	0.085	2.315	0.890 – 6.023
LAFB before TAVI	0.367	0.509	0.117 – 2.205
**General procedural characteristics**			

Comment [MR31]: space was added

Hybrid operation room	**0.028**	0.408	0.184 – 0.907
Procedure duration (min)	**< 0.001**	1.014	1.008 – 1.020
Laboratory time (min)	**< 0.001**	1.011	1.005 – 1.016
Contrast medium consumption (ml)	**0.015**	1.006	1.001 – 1.010
Fluoroscopy time (min)	**0.047**	1.058	1.001 – 1.120
Radiation dose ($cGycm^2$)	0.263	1	1.000 – 1.000
**Route access**			
Transfemoral	0.303	0.652	0.288 – 1.473
Transapical	0.303	1.535	0.679 – 3.467
Valve types			
Edwards valve	**0.038**	2.464	1.050 – 5.779
Medtronic valve	0.219	0.557	0.219 – 1.416
Symetis valve	0.218	0.399	0.092 – 1.722
Sheath size	0.753	1.109	0.583 – 2.111
Valve size	0.239	0.87	0.690 – 1.097
**Specific procedural characteristics**			
Predilatation	0.118	2.101	0.828 – 5.332
Rapid pacing	0.3	1.693	0.625 – 4.582
Postdilatation	0.151	0.228	0.030 – 1.713
**Postprocedural Outcomes**			
Stroke	0.447	2.275	0.274 – 18.906
Major bleeding	0.641	1.644	0.204 – 13.240
Minor bleeding	0.832	0.802	0.104 – 6.192
Blood transfusion	**< 0.001**	9.606	3.929 – 23.487

Acute kidney injury (all stages)	**0.001**	4.064	1.729 – 9.548
AKIN I	0.638	0.808	0.332 – 1.965
AKIN III	< **0.001**	19.239	7.694 – 48.106
Major vascular complication	< **0.001**	7.871	2.802 – 22.110
Minor vascular complication	0.42	0.435	0.057 – 3.296
New AV block 1	0.709	0.755	0.173 – 3.300
New AV block 3	0.913	1.086	0.246 – 4.795
New LBBB	0.187	0.439	0.129 – 1.492
New RBBB	0.244	3.664	0.412 – 32.557
New LAFB	0.879	1.122	0.254 – 4.963
Hemodynamic relevant arrhythmias	< **0.001**	14.605	5.857 – 36.423
New permanent pacemaker implantation	0.529	1.383	0.504 – 3.792
Paravalvular insufficiency	0.669	0.749	0.200 – 2.810
Hospitalization after TAVI (days)	**0.044**	1.013	1.000 – 1.025

Comment [MR32]: space was added

Supplementary Figure 1: **Development of annual TAVI procedures at Heart Center Fulda.** Description: Group 1 (years 2010-2015), Group 2 (years 2016-2017) and Group 3 (years 2018-2019).

Comment [s33]: Name of the table must be fat.

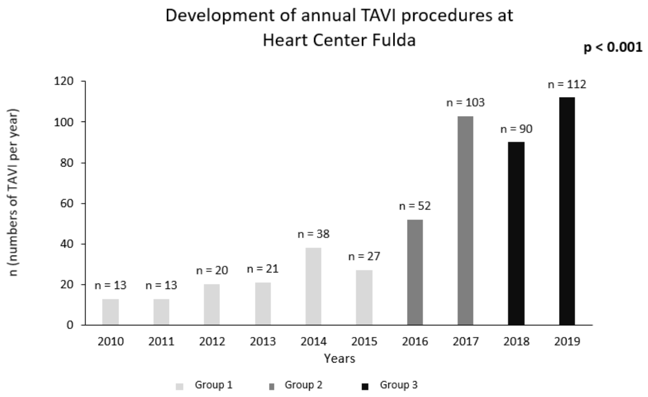

Development of annual TAVI procedures at Heart Center Fulda
p < 0.001
n (numbers of TAVI per year)
120
100
80
60
40
20
0
n = 13
n = 13
n = 20
n = 21
n = 38
n = 27
n = 52
n = 103
n = 90
n = 112
2010
2011
2012
2013
2014
2015
2016
2017
2018
2019
Years
Group 1
Group 2
Group 3
